# Cross-sectional study on the association between 24-hour urinary potassium excretion and the risk of H-type hypertension and non-H-type hypertension in Chinese adults

**DOI:** 10.3389/fendo.2025.1522607

**Published:** 2025-06-23

**Authors:** Qingfang He, Xiaofu Du, Xiangyu Chen, Lixin Wang, Yujia Fang, Jieming Zhong

**Affiliations:** Department of Chronic Non-Communicable Diseases Control and Prevention, Hangzhou, China

**Keywords:** 24-h urinary potassium excretion, H-type hypertension, non-H-type hypertension, homocysteine, diet, knowledge attitude and behavior

## Abstract

**Objective:**

Reports on urinary potassium excretion and H-type hypertension remains rare. We aimed to describe the relationship between 24-hour (h) urinary potassium excretion and the risk of H-type hypertension and non-H-type hypertension, thus to guide personal and public health campaigns including dietary recommendations to lower H-type hypertension and non-H-type hypertension in China.

**Methods:**

In 2020 in Zhejiang, China, a cross-sectional survey on Salt Reduction and Prevention of Hypertension was conducted by using a stratified multistage random sampling strategy, with participants aged 21 to 72 years. Standardized questionnaires, physical examinations and laboratory measurements were carried out among them, as well as survey on knowledge, attitudes, and behavior (KAB) correlated with salt and hypertension. 24-h urine specimens were collected for sodium and potassium excretion measurement. Subjects were divided into three groups, normal blood pressure, non-H-type hypertension and H-type hypertension, according to their blood pressure, self-reported history of hypertension and blood homocysteine (Hcy) levels.

**Results:**

1141 participants with complete information were obtained with the median age of 53 years, 46.0% were males, 41.7% were hypertension, among which 80.5% were H-type hypertension. The median Hcy and 24-h urinary potassium were 11.7μmol/L and 40.83 mmol/24h, respectively. After controlling for confounding factors, logistic regression analysis displayed that for every 1 mmol/L increase in 24-h urinary potassium excretion, the risk of H-type hypertension and non-H-type hypertension decreased by 1.4% (OR=0.986, 95% CI: 0.978-0.995, *P*=0.002) and 1.7% (OR=0.983, 95% CI: 0.968-0.998, *P*=0.025), respectively. Restricted cubic spline (RCS) curves showed that with the increase of 24-h urinary potassium excretion, the risk of both H-type hypertension (*P*= 0.008) and non-H-type hypertension (*P*=0.027) decreased, while there were no nonlinear dose-response relationships between 24-h urinary potassium excretion with both H-type hypertension (*P* for non-linearity = 0.881) and non-H-type hypertension (*P* for non-linearity = 0.101). Participants’ level of KAB in H-type hypertension group presented a lower percentage (86.2%) knowledge of the risk factors of hypertension, a highest percentage (12.5%) of self-assessment of excessive salt intake, but a lowest percentage (23.0%) of taking initiative to reduce salt intake.

**Conclusion:**

24-h urinary potassium excretion was negatively associated with the risk of both H-type hypertension and non-H-type hypertension, with a more stable negative response observed in H-type hypertension across logistic models and RCS curves. It is recommended that hypertension particularly H-type hypertension patients strengthen their KAB regarding salt reduction and hypertension, adopt reasonable dietary patterns, to improve potassium intake, and ultimately aid in better Hcy and blood pressure management.

## 1 Introduction

H-type hypertension, defined as hypertension combined with elevated homocysteine (Hcy) level (≥10 μmol/L), was first proposed by Chinese scholars considering its high prevalence and potential harm, and they developed the Chinese Expert Consensus (1, 2). Hcy, generated by the demethylation of methionine in liver, muscle and other tissues, is a sulfhydryl amino acid. Enzymatic deficiencies or reduced activity in Hcy metabolism, deficiencies in folic acid, vitamin B6, or vitamin B12, and unhealthy lifestyles, are positively correlated with elevated Hcy levels (3). Influenced by population genetic characteristics, diet and other factors, H-type hypertension is widely prevalent in Chinese hypertension patients (approximately 75%) (4). Domestic and foreign guidelines for the prevention and treatment of hypertension emphasize that reasonable diet is an important and effective means to prevent and treat hypertension. Furthermore, diets rich in folic acid, vitamin B12, vitamin B6 and other antioxidant nutrients have a good effect on reducing Hcy level in the population. Compared with singly controlling blood pressure, coupled with folic acid supplementation to lower the Hcy level of blood can further lower the risk of stroke in patients with hypertension in China, and the effect is more significant in hypertension with increased blood Hcy level (5). Therefore, the control of H-type hypertension is one of the most important strategies to deal with the high incidence of stroke in China (6).

Dietary potassium’s intake displays a close relationship to the development of hypertension. Studies have shown that the increase in the intake of potassium-rich vegetables and fruits or potassium salts in the early stage can promote sodium excretion and vasodilation, and further reduce blood pressure (7), which is an important non-drug means to prevent and treat hypertension and its complications. Meanwhile, dietary approaches to stop hypertension (DASH) with low sodium and high potassium have been shown to lower blood homocysteine levels and improve the efficacy of antihypertensive drug therapy (8). Therefore, it is meaningful to explore the relationship between potassium and H-type hypertension and non-H-type hypertension. Though it is difficult to assess the amount of sodium and potassium intake in daily diet, some studies have shown that they can be evaluated by the 24-hour urinary electrolyte levels (9). However, there are few reports on the correlation between potassium intake and homocysteine, H-type hypertension and non-H-type hypertension.

In this context, in 2020 a cross-sectional survey was performed in Zhejiang, China, with the objectives of exploring the potential risk factors for H-type hypertension and non-H-type hypertension, investigating the correlation between 24-h urinary potassium excretion and the risk of these two hypertension subtypes, and analyzing the associations between hypertension subtypes and salt/hypertension related knowledge, attitudes, and behavior (KAB). These findings are expected to provide a theoretical basis for the personalized prevention and control strategies, as well as targeted dietary interventions, for H-type hypertension and non-H-type hypertension.

## 2 Materials and methods

### 2.1 Research content and methodology

**Figure 1:**
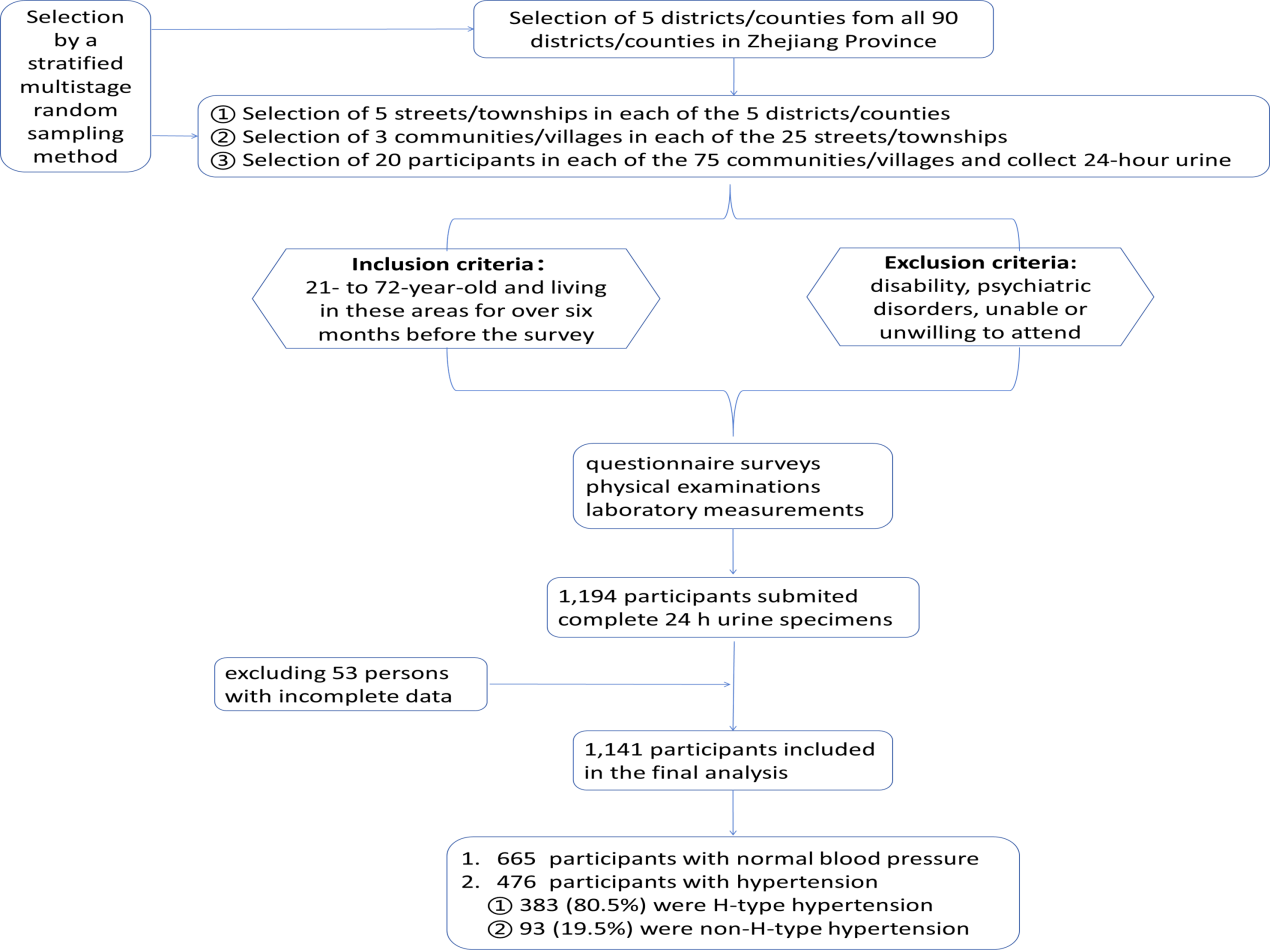
The flowchart illustrating the selection criteria and the research process.

In 2020 a cross-sectional survey on Salt Reduction and Prevention of Hypertension was performed in Zhejiang Province, China. As shown in **Figure 1**, totally 2 rural areas and 3 urban areas in northeastern, eastern, middle western, southern and central Zhejiang Province were included. By a stratified multistage randomized sampling method, subjects of 21- to 72-year-old and living in these areas for over six months before the survey, excluding disability/psychiatric disorders/unable or unwilling to attend, were randomly selected. The Ethical Review Committee of Zhejiang Provincial Center for Disease Control and Prevention approved the survey(ID. 2019056), and all the subjects signed informed consent forms.

Face to face questionnaires, physical examination, and laboratory tests were combined to collect the information including demographic characteristics, lifestyle factors including smoking, drinking, dietary patterns, physical activities, history of hypertension, as well as physical examination results such as blood pressure, weight, height, waist circumference (WC), and hip circumference (HC).

Overnight fasting venous blood specimens were obtained to measure fasting blood glucose (FBG), triglycerides (TG), total cholesterol (TC), high density lipoprotein cholesterol (HDL-C), homocysteine (Hcy), low density lipoprotein cholesterol (LDL-C), uric acid (UA), creatinine (CREA), UREA, C reactive protein, serum K^+^ and Na^+^. Enzymatic method was used to measure TC, TG, Hcy and CREA, direct clearance method to measure HDL-C and LDL-C, UA by the uricase Uv method, UREA by the Urease UV rate method, K^+^ and Na^+^ by the ion selective electrode method, and an Abbott C16000 biochemical instrument (,Abbott Corp., Chicago, IL, USA) was used. C reactive protein was measured by the immunity transmission turbidity method(C501, Roche Cobas Corp., Basel, Switzerland).

24-h urine specimens were collected for urinary potassium and sodium excretion, microalbuminuria and creatinine analysis. With Abbott C16000 (Abbott Corp., Chicago, IL, USA), urinary potassium and sodium were measured by the ion selective electrode method while urinary creatinine by the picric acid method. With Roche C501 (Roche Cobas Corp., Basel, Switzerland) urinary microalbuminuria were measured by the immunity transmission turbidity method. Urinary excretion equals to the cross-product of the 24-h urine volume multiplied by the concentration of the analyte. Blood and 24-h urine specimens were all measured by Hangzhou KingMed Diagnostics Co., LTD.

Participants’ KAB related to salt and hypertension were collected and totally 19 questions were selected.

The measurements methods of blood pressure, weight, height, HC and WC were the same as literature (10, 11). The percentage of body fat was measured with a Omron (OMRON, Yangzhou, China) body fat scales, which is accurate to 0.1%.

### 2.2 Definition and grouping

According to 2018 Chinese guidelines for prevention and treatment of hypertension (12), the standard of hypertension refers to: self-reported use of antihypertensive medication within two weeks, and/or diastolic blood pressure (DBP) ≥ 90 mmHg, and/or systolic blood pressure (SBP) ≥ 140 mmHg.

H-type hypertension refers to: hypertension combined with blood Hcy level ≥ 10 μmol/L (1). Non-H-type hypertension refers to: hypertension combined with blood Hcy level < 10 μmol/L.

Body mass index (BMI) equals to weight (kg)/(height (m) ^2^).

Current smoking refers to those who smokes more than 1 cigarette per day cumulatively or consecutively over 6 months.

Alcohol drinking refers to a response ≥1 time per week during the past year. Alcoholic beverages included liquor, beer, rice wine, and red wine.

Self-reported physical activity refers to ≥75 minutes per week of high-intensity exercise, or ≥150 minutes per week of moderate-intensity or a combination of high- and moderate-intensity exercise.

Dietary patterns was divided into three groups, that is, vegetarian diet, balanced intake of vegetables and meat, and meat diet, based on their answers to the question “Compared to others, which is the main part of your personal diet: vegetarian (vegetables, soy products, etc.), balanced intake of vegetables and meat, or meat (meat, eggs, animal offal, etc.)?”.

### 2.3 Quality control

All investigators are health professionals and have been trained uniformly and qualified. Quality control team including both the county (city) CDCs and provincial CDC was established. The survey data was entered by tablet computer on site, and the provincial inspectors were responsible for spot checking 5% of the questionnaire, and judged the completeness and logical errors of the questionnaire by listening to the field recordings.

### 2.4 Statistical analysis

The abnormal distribution continuous variables were denoted by M (Q1, Q3), and the comparison among different groups was performed by non-parametric tests (such as rank-sum test). The categorical data were denoted as frequency (n) and percentage (%). χ^2^ test and Wilcoxon rank sum test were used for group comparisons of categorical and rank variables, respectively. Logistic regression was adopted to analyze the influence factors of different types of hypertension, as well as the effect of 24-h urinary potassium excretion level on the risk of non-H-type and H-type hypertension. Restricted cubic spline (RCS) curve was used to analyze the dose-response relationship between 24-h urinary potassium excretion with H-type hypertension and Non-H-type hypertension (The reference value for 24-h urinary potassium excretion was the median overall 24-h urinary potassium of 40.83 mmol/24h, Knot =3). SAS software (SAS Institute Inc., Cary, NC, USA, Version 9.4) and R version 4.3.0 (http://www.R-project.org) were used. P values < 0.05 were regarded as statistically significant.

## 3 Results

### 3.1 Characteristics of different types of hypertension

1,194 participants submitted complete 24-h urine specimens. After excluding 53 persons with incomplete data, finally 1,141 subjects were obtained. As listed in **Table 1**, the median age of the 1,141 subjects was 53 (40, 63) years, 46.0% were males. The percentage of <9, 9–12, and ≥12 years of education were 34.3%, 47.0%, and 18.8% respectively. The median DBP and SBP were 78 mmHg and 128 mmHg, respectively. The median Hcy and 24-h urinary potassium were 11.7 (9.4, 15.0) μmol/L and 40.83 (28.24, 56.40) mmol/24h, respectively.

**Table 1:** Participants’ characteristics of different types of hypertension.

Characteristics	All (n=1141)	Normal blood pressure (n=665)	Non-H-type hypertension (n=93)	H-type hypertension (n=383)	*χ^2^/H*	*P* value
Age [years, *M*(*Q*_1_,*Q*_3_)]	53 (40, 63)	45 (35, 57)	56 (50, 60)	62 (53, 68)	206.36	0.000**
Gender, *n(%)*					54.10	0.001**
Male	525 (46.0)	288 (43.3)	16 (17.2)	221 (57.7)		
Female	616 (54.0)	377 (56.7)	77 (82.8)	162 (42.3)		
Education years, *n(%)*					111.52	0.000**
<9	391 (34.3)	170 (25.6)	38 (40.9)	183 (47.8)		
9–12	536 (47.0)	308 (46.3)	48 (51.6)	180 (47.0)		
≥ 12	214 (18.8)	187 (28.1)	7 (7.5)	20 (5.2)		
Smoking, *n(%)*	258 (22.6)	129 (19.4)	7 (7.5)	122 (31.9)	34.71	0.000**
Drinking, *n(%)*	278 (24.4)	134 (20.2)	22 (23.7)	122 (31.9)	18.09	0.000**
Dietary patterns, *n(%)*					21.80	0.000**
Vegetarian diet	272 (23.8)	135 (20.3)	30 (32.3)	107 (27.9)		
Balanced intake of vegetables and meat	772 (67.7)	476 (71.6)	62 (66.7)	234 (61.1)		
Meat diet	97 (8.5)	54 (8.1)	1 (1.1)	42 (11.0)		
Regular physical activity, *n(%)*	553 (48.5)	312 (46.9)	59 (63.4)	182 (47.5)	9.13	0.010*
BMI [kg/m^2^, *M*(*Q*_1_,*Q*_3_)]	23.8 (21.8, 26.0)	23.1 (21.3, 25.2)	24.6 (22.5, 27.1)	24.9 (22.7, 27.3)	67.33	0.000**
WC [cm, *M*(*Q*_1_,*Q*_3_)]	83.0 (77.0, 89.5)	81.0 (75.0, 87.0)	84.5 (78.2, 90.0)	86.7 (81.0, 93.0)	86.129	0.000**
HC [cm, *M*(*Q*_1_,*Q*_3_)]	94.0 (89.6, 98.5)	93.0 (89.0, 97.0)	95.0 (89.8, 100.0)	95.0 (91.0, 100.0)	34.125	0.000**
Percentage of body fat [%, *M*(*Q*_1_,*Q*_3_)]	29.6 (25.1, 34.0)	28.7 (24.3, 32.8)	33.2 (28.4, 36.2)	30.1 (25.7, 35.2)	41.63	0.000**
SBP [mmHg, *M*(*Q*_1_,*Q*_3_)]	128 (117, 141)	120 (111, 128)	144 (138, 151)	144 (134, 154)	590.80	0.000**
DBP [mmHg, *M*(*Q*_1_,*Q*_3_)]	78 (72, 86)	74 (70, 80)	88 (81, 93)	86 (78, 92)	334.01	0.000**
HCY [μmol/L, *M*(*Q*_1_,*Q*_3_)]	11.7 (9.4, 15.0)	10.8 (8.8, 13.7)	9.0 (8.2, 9.4)	14.1 (11.8, 17.6)	288.03	0.000**
FBG [mmol/L, *M*(*Q*_1_,*Q*_3_)]	4.88 (4.53, 5.46)	4.75 (4.41, 5.20)	5.01 (4.59, 5.92)	5.09 (4.70, 5.92)	78.39	0.000**
TC [mmol/L, *M*(*Q*_1_,*Q*_3_)]	4.74 (4.11, 5.35)	4.66 (410, 5.26)	5.06 (4.21, 5.48)	4.76 (4.11, 5.43)	6.32	0.042*
TG [mmol/L, *M*(*Q*_1_,*Q*_3_)]	1.42 (1.05, 2.02)	1.31 (0.95, 1.89)	1.48 (1.10, 2.24)	1.60 (120, 2.24)	37.49	0.000**
HDLC [mmol/L, *M*(*Q*_1_,*Q*_3_)]	1.40 (1.19, 1.64)	1.42 (1.22, 1.66)	1.44 (1.24,1.69)	1.34 (1.16,1.58)	14.21	0.001**
LDLC [mmol/L, M(Q1,Q3)]	2.51 (2.03, 3.06)	2.48 (2.02, 3.01)	2.59 (2.09, 3.08)	2.56 1.99, 3.13)	1.72	0.423
UA [μmol/L, *M*(*Q*_1_,*Q*_3_)]	306 (258, 369)	291 (245, 356)	291 (255, 350)	332 (289, 391)	65.20	0.000**
CREA [mmol/L, M(Q1,Q3)]	62 (54, 74)	60 (52, 72)	56 (49, 60)	68 (58, 80)	97.47	0.000**
UREA [mmol/L, M(Q1,Q3)]	4.8 (4.0, 5.7)	4.6 (3.9, 5.5)	4.8 (4.0, 5.6)	5.1 (4.2, 6.1)	38.07	0.000**
C reactive protein [mg/L, *M*(*Q*_1_,*Q*_3_)]	1.4 (0.9, 2.1)	1.4 (0.8, 2.1)	1.3 (0.9, 2.0)	1.4 (0.9, 2.3)	1.96	0.375
Serum K^+^ [mmol/L, M(Q1,Q3)]	4.31 (4.03, 4.66)	4.31 (4.04, 4.65)	4.28 (4.07, 4.63)	4.31 (4.02, 4.69)	0.22	0.894
Serum Na^+^ [mmol/L, M(Q1,Q3)]	140 (139, 142)	140 (139, 141)	140 (139, 141)	141 (139, 142)	9.93	0.007**
24-h urinary sodium [mmol/24h, M(Q1,Q3]	150 (106, 204)	153 (110, 206)	152 (96, 201)	146 (98, 204)	4.02	0.134
24-h urinary potassium [mmol/24h, M(Q1,Q3]	40.83 (28.24, 56.40)	42.41 (28.77, 59.49)	41.72 (31.07, 53.64)	38.18 (26.74, 54.83)	6.57	0.037*
24-h urinary sodium-to-potassium ratio [mmol/mmol, M(Q1,Q3]	3.74 (2.70, 5.10)	3.79 (2.68, 5.06)	3.63 (2.70, 4.74)	3.73 (2.75, 5.30)	1.07	0.585
24-h urinary creatinine [mmol/24h, M(Q1,Q3]	6.72 (4.95, 9.52)	6.80 (4.96, 9.66)	6.56 (4.99, 8.53)	6.61 (4.88, 9.23)	2.05	0.359
24-h urinary microalbuminuria [mg/24h, M(Q1,Q3]	8.80 (6.05, 11.56)	8.36 (5.70, 10.52)	10.61 (7.88, 12.66)	9.79 (6.73, 13.04)	38.32	0.000**

*significance level<0.05; **significance level<0.01.

Of the 1,141 participants, 476 (41.7%) were hypertension, among which 383 (80.5%) were H-type hypertension and 93 (19.5%) were non-H-type hypertension. H-type hypertension were more likely to be older, men, low education level, drinker, current smoker and meat diet. H-type hypertension had significantly higher SBP, Hcy, BMI, WC, HC, FBG, TG, UA, CREA, UREA, serum Na^+^, and significantly lower HDLC and 24-h urinary potassium. Non-H-type hypertension had significantly higher percentage of body fat, DBP, TC and 24-h urinary microalbuminuria, but significantly lower Hcy and CREA. There were no difference of 24-h urinary sodium among the normal blood pressure group, non-H-type hypertension group and H-type hypertension group.

### 3.2 Influence factors of different types of hypertension

**Figure 2:**
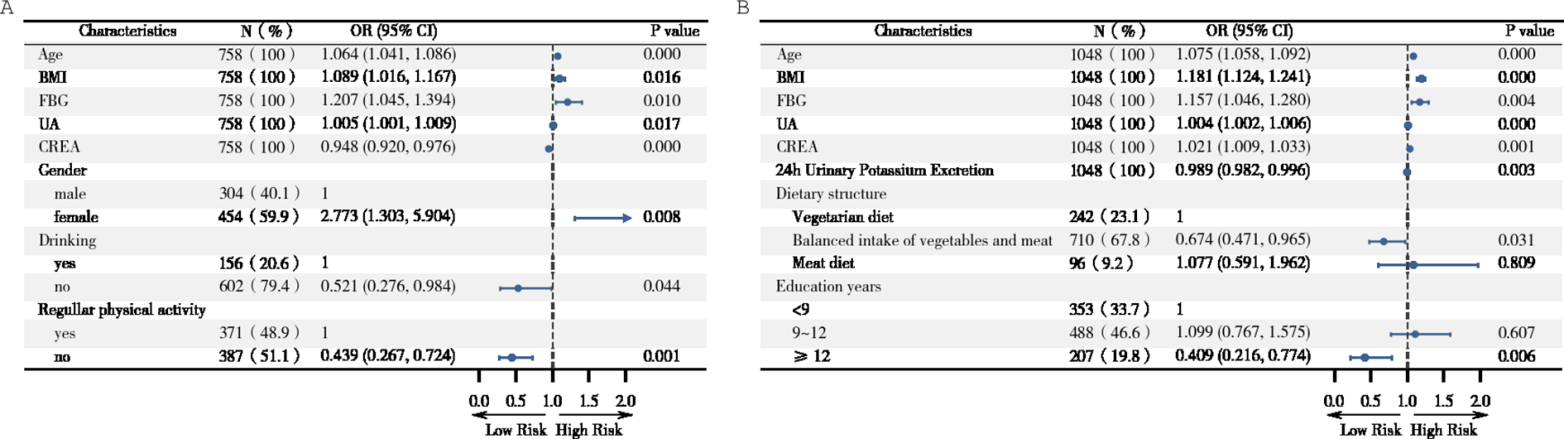
Forest plot of influence factors of different type of hypertension. **(A)** odds ratio (*OR*) with 95% confidence interval (*CI*) in selected subgroups of Non-H-type hypertension and normal blood pressure. **(B)** *OR* (95% *CI*) in selected subgroups of H-type hypertension and normal blood pressure. Factors included in the analysis were age (continues), gender (male = 1, female = 2), education years (<9 years = 1, 9–12 years = 2, ≥12 years = 3), smoking (yes=1, no=2), drinking (yes=1, no=2), dietary patterns (vegetarian diet = 1, balanced intake of vegetables and meat = 2, meat diet = 3), regular physical activity (yes=1, no=2), BMI (continues), WC (continues), HC (continues), percentage of body fat (continues), FBG (continues), TC (continues), TG(continues), HDLC(continues), LDLC (continues), UA (continues), UREA (continues), CREA(continues), C reactive protein (continues), serum K^+^(continues), serum Na^+^(continues), 24-h urinary sodium (continues), 24-h urinary potassium (continues), 24-h urinary sodium-to-potassium ratio (continues), 24-h urinary creatinine (continues), and 24-h urinary microalbuminuria (continues).

When performing logistic regression analyses, we took the normal blood pressure group as the control group, the H-type hypertension group and non-H-type hypertension group respectively as the dependent variable, and independent variables were gender (male = 1, female = 2), education years (<9 years = 1, 9–12 years = 2, ≥12 years = 3), current smoking (yes=1, no=2), drinking (yes=1, no=2), dietary patterns (vegetarian diet = 1, balanced intake of vegetables and meat = 2, meat diet = 3), regular physical activity (yes=1, no=2), and continues variables: age, BMI, WC, HC, percentage of body fat, FBG, TC, TG, HDLC, LDLC, UA, UREA, CREA, C reactive protein, serum K^+^, serum Na^+^, 24-h urinary sodium, 24-h urinary potassium, 24-h urinary sodium-to-potassium ratio, 24-h urinary creatinine and microalbuminuria. The forest plot (**Figure 2**) showed that age, BMI, FBG, UA and gender(female) were positively associated with the risk of non-H-type hypertension, while CREA, non-drinking and non-regular physical activity were negatively associated with the risk of non-H-type hypertension. Age, BMI, FBG, UA and CREA were significantly positively associated with the risk of H-type hypertension, while dietary patterns (balanced intake of vegetables and meat),24-h urinary potassium and education years (≥12 yrs) were significantly negatively associated with the risk of H-type hypertension.

### 3.3 Correlation analysis between 24-h urinary potassium excretion and the risk of different types of hypertension

When performing logistic regression analyses, we took the normal blood pressure group as the control group, and the H-type hypertension group and non-H-type hypertension group respectively as the dependent variable, and 24-h urinary potassium excretion as the independent variable. After adjusting for confounding variables (gender (male = 1, female = 2), education years (<9 years = 1, 9–12 years = 2, ≥12 years = 3), current smoking (yes=1, no=2), drinking (yes=1, no=2), dietary patterns (vegetarian diet = 1, balanced intake of vegetables and meat = 2, meat diet = 3), regular physical activity (yes=1, no=2), and continues variables: age, BMI, WC, HC, percentage of body fat, FBG, TC, TG, HDLC, LDLC, UA, UREA, CREA, C reactive protein, serum Na^+^, 24-h urinary sodium, 24-h urinary creatinine, 24-h urinary microalbuminuria), the results displayed that the risk of H-type hypertension was significantly negatively associated with 24-h urinary potassium excretion in all the subjects (*OR*=0.986, 95% *CI*: 0.978-0.995, *P*=0.002), male subjects (*OR*=0.987, 95% *CI*: 0.975-0.999, *P*=0.029) and female subjects (*OR*=0.983, 95% *CI*: 0.969-0.997, *P*=0.019); while the risk of non-H-type hypertension was significantly negatively associated with 24-h urinary potassium excretion in all the subjects (*OR*=0.983, 95% *CI*: 0.968-0.998, *P*=0.025) and male subjects (*OR*=0.924, 95% *CI*: 0.857-0.995, *P*=0.038), whereas no significant association was found in female subjects (*P*>0.05*)* (**Table 2**). Specifically, for every 1 mmol/L increase in 24-h urinary potassium excretion, the risk of H-type hypertension and non-H-type hypertension decreased by 1.4% and 1.7%, respectively. In male subjects, the risk of non-H-type hypertension was further reduced by 7.6% with each 1 mmol/L increase in urinary potassium excretion.

**Table 2:** Logistic regression analysis on the correlation of 24-h urinary potassium excretion with the risk of H-type hypertension and Non-H-type hypertension.

Groups/Models	H-type hypertension		Non-H-type hypertension	
	*OR* (95% *CI*)	*P* value	*OR* (95% *CI*)	*P* value
All	*n*=1048		*n*=758	
Model 0	0.992 (0.987-0.998)	0.006**	0.996 (0.986-1.005)	0.370
Model 1	0.992 (0.985-0.998)	0.010*	0.992 (0.981-1.003)	0.139
Model 2	0.993 (0.986-0.999)	0.034*	0.991 (0.980-1.002)	0.103
Model 3	0.989 (0.982-0.996)	0.003**	0.989 (0.978-1.001)	0.071
Model 4	0.986 (0.978-0.995)	0.002**	0.983 (0.968-0.998)	0.025*
Male	*n*=509		*n*=304	
Model 0	0.994 (0.986-1.002)	0.116	0.996 (0.975-1.018)	0.708
Model 1	0.992 (0.984-1.001)	0.068	0.994 (0.972-1.016)	0.578
Model 2	0.994 (0.986-1.003)	0.205	0.991 (0.968-1.015)	0.469
Model 3	0.990 (0.981-0.999)	0.036**	0.991 (0.967-1.016)	0.467
Model 4	0.987 (0.975-0.999)	0.029*	0.924 (0.857-0.995)	0.038*
Female	*n*=539		*n*=454	
Model 0	0.992 (0.984-1.000)	0.060	0.993 (0.982-1.004)	0.225
Model 1	0.991 (0.982-1.001)	0.085	0.991 (0.979-1.004)	0.171
Model 2	0.992 (0.982-1.002)	0.107	0.991 (0.978-1.004)	0.191
Model 3	0.989 (0.978-0.999)	0.036*	0.990 (0.976-1.004)	0.145
Model 4	0.983 (0.969-0.997)	0.019*	0.990 (0.973-1.007)	0.224

Model 0: Crude *OR* (95% *CI*).

Model 1: For all, adjusted for age, gender; for male or female group, adjusted for age;

Model 2: Model 1 + Adjusted for education years, smoking, drinking, dietary patterns, regular physical activity.

Model 3: Model 2 + Adjusted for BMI, WC, HC, percentage of body fat;

Model 4: Model 3 + Adjusted for FBG, TG, CHOL, HDLC, LDLC, UA, CREA, UREA, C reactive protein, serum Na^+^, 24-h urinary sodium, 24-h urinary creatinine, 24-h urinary microalbuminuria.

*significance level<0.05; **significance level<0.01.

**Figure 3:**
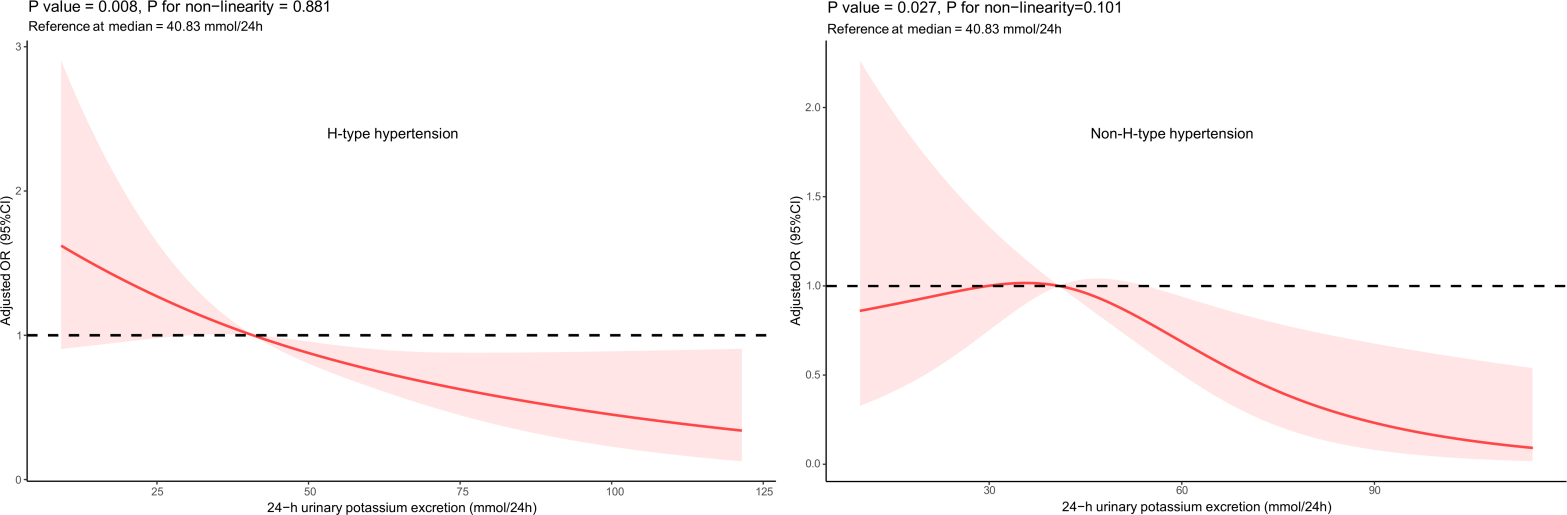
Adjusted Odds Ratio (OR) (95% CI): After adjusting for age, gender, education years, current smoking, drinking, dietary structure, regullar physical activity, BMI, WC, HC, percentage of body fat, FBG, TC , TG, HDLC, LDLC, UA, UREA, CREA, C reactive protein,serum Na+, 24 h urinary sodium, 24 h urinary creatinine, 24 h urinary microalbuminuria

Plot RCS with the reference of the median 24-h urinary potassium excretion(40.83 mmol/24h). After adjusting for age, gender, education years, current smoking, drinking, dietary patterns, regular physical activity, BMI, WC, HC, percentage of body fat, FBG, TC, TG, HDLC, LDLC, UA, UREA, CREA, C reactive protein, serum Na^+^, 24-h urinary sodium, 24-h urinary creatinine, 24-h urinary microalbuminuria, RCS curves showed that with the increase of 24-h urinary potassium excretion, the risk of both H-type hypertension (*P* = 0.008) and non-H-type hypertension (*P* =0.027) decreased. There was no non-linear dose-response relationship between 24-h urinary potassium excretion with both H-type hypertension (*P* for non-linearity = 0.881) and non-H-type hypertension (*P* for non-linearity=0.101) (**Figure 3**).

### 3.4 Knowledge, attitude and behavior

As listed in **Table 3**, more than two thirds of the participants had the hypertension related knowledge, knew the hazards of high-salt diets and who needs a low-salt diet, but only 58.1%, 43.3%, 34.9% had the knowledge of the recommendation of less than 6 g salt intake daily, low-sodium salt and low-sodium salt reduced blood pressure, respectively. Most participants expressed an approval attitude to labeling sodium/salt content in processed foods or adopting low-salt diets.

**Table 3:** Comparison of knowledge, attitude, behavior grouped by types of hypertension.

Characteristics	All (n=1141)	Normal blood pressure (n=665)	Non-H-type hypertension (n=93)	H-type hypertension (n=383)	*P value*
	*n* (%)				
Knowledge					
Know the diagnostic criteria of hypertension	754 (66.1)	428 (64.4)	64 (68.8)	262 (68.4)	0.348
Know the hazards of hypertension	979 (85.8)	557 (86.8)	83 (89.2)	319(83.3)	0.183
Know the risk factors of hypertension	1022 (89.6)	612 (92.0)	80 (86.0)	330 (86.2)	0.006**
Know salt restriction 6 grams per day per person	663 (58.1)	411(61.8)	53 (57.0)	199 (52.0)	0.008**
Know low-salt diet helps lower blood pressure	930 (81.5)	535 (80.5)	82 (88.2)	313 (81.7)	0.197
Know the hazards of high-salt diet	992 (86.9)	587 (88.3)	82 (88.2)	323 (84.5)	0.178
Know who needs a low-salt diet	1056 (93.6)	621 (93.4)	84 (90.3)	351 (91.6)	0.408
Know low-sodium salt	494 (43.3)	322 (48.4)	41 (44.1)	131 (34.2)	0.000**
Know low-sodium salt helps control blood pressure	398 (34.9)	255 (38.3)	35 (37.6)	108 (28.2)	0.003**
Attitude					
Approve of promoting low-salt diet	1070 (93.8)	625 (94.0)	86 (92.5)	359 (93.7)	0.852
Approve of low-salt diet	1051 (92.1)	609 (91.6)	84 (90.3)	358 (93.5)	0.439
Approve of labeling sodium/salt content in processed foods	875 (76.7)	549 (82.6)	70 (75.3)	256 (66.8)	0.000**
Believe that labeling sodium/salt content in processed foods helps to choose low-salt foods	875 (76.7)	543 (81.7)	69 (74.2)	263 (68.7)	0.000**
Behavior					
Self-assessment of salt intake					0.009**
A little	394 (34.5)	206 (31.0)	44 (47.3)	144 (37.6)	
Moderate	615 (53.9)	383 (57.6)	41 (44.1)	191 (49.9)	
Excessive	132 (11.6)	76 (11.4)	8 (8.6)	48 (12.5)	
Received publicity or education on low-salt diet	781 (68.4)	467 (70.2)	66 (71.0)	248 (64.8)	0.160
Attention to the sodium/salt content label in processed foods	341 (29.9)	218 (32.8)	28 (30.1)	95 (24.8)	0.025*
Plan to reduce salt intake	1019 (89.3)	586 (88.1)	86 (92.5)	347 (90.6)	0.269
Take initiative to reduce salt intake	828 (72.6)	464 (69.8)	69 (74.2)	295 (23.0)	0.038 *
Using or used low-sodium salt	229 (20.1)	151 (22.7)	12 (12.9)	66 (17.2)	0.020*

*significance level<0.05; **significance level<0.01.

Data are presented as frequency (n) and percentage (%).

As for the behavior, 11.6% participants still reported a self-assessment of excessive salt intake, 29.9% indicated that they had concerned about the sodium/salt content label in processed foods, and 20.1% had actually ever used or were using low-sodium salt.

**Table 3** also told that different types of hypertension groups differed significantly on about half the KAB questions. Overall, normal blood pressure displayed significantly better KAB than H-type hypertension and non-H-type hypertension groups. It’s worth paying attention that H-type hypertension group had a lower percentage (86.2%) knowledge of the risk factors of hypertension, the highest percentage (12.5%) of self-assessment of excessive salt intake, while the lowest percentage (23.0%) of taking initiative to reduce salt intake.

## 4 Discussion

The proportion of H type hypertension in patients with hypertension in China was reported as high as 75.0% (4). The Hcy level of hypertensive patients is about 50% higher in China than in the United States (13), and the risk of stroke in patients with hypertension is about twice that in Japan, Europe and America, which might be due to the high Hcy levels in patients of China (14). Studies have shown that the increase of Hcy level is closely related to the occurrence and development of hypertension: high Hcy activates angiotensin converting enzyme by inhibiting the production of endogenous hydrogen sulfide in the body, produces angiotensin II and acts on angiotensin type 1 receptor, thus leading to a series of pathological processes such as the increase of blood pressure and vascular proliferation (15, 16). Meanwhile, numerous researches have consistently shown an independent correlation between elevated Hcy level and all-cause mortality or cardiovascular disease. Starting at a plasma Hcy concentration of around 10 μmol/L, the increasing risk follows a linear dose-response relationship without a specific threshold level (17). Wald’s Meta-analysis revealed that for every 5 μmol/L increase in plasma Hcy, stroke risk increased by 59% (OR= 1.59; 95%CI:1.30-1.95), while lowering plasma Hcy by 3mol/L reduced stroke risk by about 24% (15%-33%) (18). Through a large sample epidemiological study, Graham et al. (3) disclosure that the incidence of cardiovascular events in H-type hypertension was nearly 5 times higher than that in hypertension only and 25–30 times higher than that in normal people. The focus of cardiovascular and cerebrovascular disease intervention is to prevent stroke. Hypertension accompanied by elevated homocysteine has a significantly synergistic effect in causing cerebrovascular and cardiovascular events, especially in stroke (3, 19). This suggests that reducing blood Hcy level while controlling hypertension has become an important strategy for the prevention and treatment of hypertension and stroke in China (6).

Although there were few reports on the potassium intake and H-type hypertension, several studies described the relationship between potassium intake and blood pressure or Hcy level. In contrast to the pressor effect of Hcy, potassium dilates blood vessels and antagonizes the pressor effect of sodium. Dietary potassium reduces blood pressure, prevents stroke, and improves cardiovascular disease. Studies have shown that, compared with a diet with K^+^ and Na^+^ intake of 1.7 g/d and 3.0 g/d, respectively, DASH diet (K^+^ and Na^+^ intake of 4.7 g/d and 3.0 g/d, respectively) could reduce diastolic and systolic blood pressure in healthy adults by 3.0 mmHg and 5.5 mmHg, respectively (20). In 2014, Mente et al. (21) selected 102,216 volunteers (42% of them from China) from low, middle and high income families of 18 countries in five continents, and tested their fasting midstream morning urine samples for urinary potassium measurement. The results showed that for every 1 g increase in potassium excretion, SBP decreased by 1.08 mmHg and DBP decreased by 0.09 mmHg, indicating that urinary potassium excretion was negatively related to blood pressure levels, and this phenomenon was more obvious in China than in other regions. Intersalt’s multicenter study on 24-hour urinary electrolyte excretion and blood pressure in 10,079 men and women aged 20–59 exhibited that potassium excretion was negatively correlated with blood pressure (22). Dietary potassium is not only negatively related to blood pressure. O’Donnell et al (23) followed 101,945 volunteers for 3.7 years and monitored 24-h urinary potassium and sodium excretion. The results showed that when the urinary potassium excretion was more than 1.5 g/24 h, with the increase of urinary potassium excretion, the cardiac mortality of hypertensive patients decreased significantly. Similarly, our logistic regression analysis showed that after controlling for confounding factors, higher 24-h urinary potassium excretion was significantly associated with a reduced risk of both H-type hypertension (*P*=0.002) and non-H-type hypertension (*P*=0.025) among all subjects.

On the other hand, methionine in foods is the direct source of Hcy in human. The content and metabolism of methionine in the body are affected by dietary patterns, vitamin nutritional status and lifestyle. It has been reported that vitamin B6, vitamin B12 and folic acid are involved in Hcy metabolism as specific coenzymes and factors, and their levels are negatively correlated with plasma Hcy levels (24, 25), and significantly lower the incidence of stroke (26, 27). Esfahani et al. (28) showed that subjects who consumed a diet rich in vegetables and fruits had significantly increased serum antioxidant vitamins, while Hcy levels were lower. Adherence to the DASH diet has been displayed not only to lower blood pressure and plasma Hcy levels (8), but also to increase urinary potassium excretion with the correlation coefficient r = 0.42 (P < 0.0001), suggesting that the DASH diet provided a high potassium intake (29). Shanghai Suburban Adult Cohort and Biobank(SSACB) study, including 38,273 participants aged 20 to 74 years, showed that the higher the intake of potassium, calcium, etc, the lower the blood Hcy level (30). Zhaofei Wan et al. found that normotensive salt-sensitive subjects’ plasma Hcy was ameliorated by potassium supplementation (34.4 ± 17.0 μmol/L versus 20.9 ± 10.4 μmol/L, *P* < 0.01) (31). Our forest plot results indicated that dietary patterns (balanced intake of vegetables and meat) and 24-h urinary potassium were significantly negatively associated with the risk of H-type hypertension while not with non-H-type hypertension. Moreover, though after adjusting for numerous confounding factors, the RCS curves displayed significant linear negative associations between 24-h urinary potassium excretion and the risks of both H-type hypertension and non-H-type hypertension, the former exhibited a relatively stable negative response in both RCS curves and logistic models. Therefore, given the current state of knowledge and the limitations of the present study, although the underlying metabolic pathways are not well understood, it is likely that patients with H-type hypertension are more sensitive to the diet - potassium excretion - Hcy metabolism - blood pressure regulation pattern. In contrast, non-H-type hypertension might be governed by other blood pressure - regulating mechanisms, which renders the effect of urinary potassium excretion less pronounced. Further research, including prospective studies and long-term follow-up studies, as well as the analysis of the integration of dietary and genetics data, are highly warranted to explore the potential mechanism.

H-type hypertension differs significantly between the genders. A survey conducted in six cities in China (Beijing, Shenyang, Harbin, Nanjing, Shanghai and Xi’an) (4) showed that the proportion of patients with H-type hypertension in Chinese hypertension was 75.0% (91% in males and 63% in females). In our study, the median plasma Hcy level in males was 13.7μmol/L, which was significantly higher than the level in females (10.2μmol/L). The proportion of H-type hypertension was as high as 80.5% (93.2% in men and 67.8% in women), which was close to the reported baseline data of 80.3% in China Stroke Primary Prevention Trial (CSPPT) (5). The difference between the genders might be related to differences in hormone levels (32), mainly considering that estrogen can significantly increase the transamination of methionine and promote the metabolic circulation of Hcy, thereby reducing the level of plasma Hcy (33). In addition, living habits between men and women such as smoking (34), drinking (35) and diet also affect the level of plasma Hcy. Long-term alcohol consumption can cause a decrease in the activity of methionine synthase in hepatocytes and abnormal metabolism of plasma Hcy, thus resulting in high Hcy, but there are also relevant reports suggesting that there is no relationship between plasma Hcy level and alcohol consumption (36). After controlling the confounding factors, our regression analysis results displayed that alcohol consumption was not associated with H-type hypertension, but had a positively association with non-H-type hypertension.

Our results displayed that H-type hypertension were significantly older than non-H-type hypertension and normotensive subjects, with the lowest percentage of ≥12 years of education. The elders often experience reduced organ function, limited chewing function, and preferences for heavy dietary taste and monotonous dietary patterns, in addition, the elderly often prefer to overcooked foods, these all resulting in the destruction and reduced absorption and utilization of nutrients. Combined with excessive intake of sodium, deficient intake of vegetables and fruits, this dietary pattern may synergistically exacerbate potassium loss, reduce B vitamins and folic acid and thus elevate Hcy and blood pressure. On the other hand, the low education level in the H-type hypertension group—coupled with limited access to health education—likely contributes to suboptimal KAB (knowledge-attitude-behavior) regarding salt intake and hypertension. As evidenced by the lower rate (86.2%) of hypertension risk factor knowledge, some H-type hypertension patients may struggle to recognize the role of dietary potassium in regulating Hcy and blood pressure, which reduces their adherence to potassium-rich diets. In H-type hypertension patients, not only did it show the highest proportion (12.5%) of self-assessment excessive salt intake, but also the lowest proportion (23.0%) actively reduced salt consumption, highlighting a critical attitude-behavior gap which exacerbates sodium-potassium homeostasis dysregulation and weakens potassium’s protective effects. This KAB disparity may disproportionately affect H-type hypertension, in which dietary factors might strongly influence homocysteine metabolism. By contrast, non-H-type hypertension’s multifactorial mechanisms (e.g., genetic or renal pathways) may dilute the observed impact of potassium. Enhancing health literacy to bridge knowledge and behavior—particularly promoting balanced diets and dietary diversity—could optimize potassium intake and amplify its protective role in H-type hypertension. Notably, while 24-h urine collection is cumbersome, measuring urinary potassium excretion remains a noninvasive, cost-effective method (costing only one-tenth to one-fifth of homocysteine or folic acid tests) for objectively monitoring potassium intake. Long-term monitoring of urinary potassium in H-type hypertension patients is therefore warranted, alongside targeted nutritional assessments, dietary interventions, and salt-reduction education. Encouraging increased consumption of vegetables, fruits, and whole grains could help meet daily potassium and folate requirements (37), maximizing intervention efficacy.

In addition, this study concluded that BMI, FBG and UA were significantly positively correlated with both H-type and non-H-type hypertension. Kim et al. reported that overweight and obesity were significantly associated with the prevalence of hypertension, with a 49% increase in the risk of developing hypertension for every 5kg/m^2^ increase in BMI (38). Molina et al. (39) found that abnormal glucose metabolism increased the risk of cardiovascular evaluation in the epidemiological study of South Asian population. A study based on Japanese community residents with hypertension showed that it was difficult to maintain the target blood pressure in those with elevated UA, so reducing UA was beneficial to prevent the progression of hypertension (40).

Our study has several limitations. Firstly, as a cross-sectional study, we can only describe associations and cannot draw causal relationships. Long-term follow-up studies are needed to demonstrate this outcome. Secondly, we only obtained the 24-h urine collection for one time, which reflects a measurement result of potassium intake in the last 1–3 days. To predict long-term average concentration, it is recommended to collect 24-h urine across multiple days to accurately assess individual potassium intake. Thirdly, small sample sizes in certain subgroups may diminish statistical power and heighten bias risk. Thus, larger-scale studies are warranted for further exploration. Lastly, methylenetetrahydrofolate reductase (MTHFR) is the key enzyme in Hcy metabolism. MTHFR gene C - > T mutation in 677 bases, especially in the lack of folic acid, leads to a direct elevation of Hcy levels. Therefore, for the dietary prevention and control of H-type hypertension, it is necessary to measure the genotype of the patients when carrying nutritional assessment and precise dietary intervention to obtain ideal results.

Our study has several strengths. It was a cross-sectional investigation with a large sample size and good representation based on community adults. All the examinations and questionnaires were measured and investigated by trained professionals in accordance with strictly standard procedures, with good authenticity. In addition, assessment of potassium excretion based upon 24-h urine specimens is considered a more objective and accurate method for estimating potassium intake. Lastly, despite limited research on the relationship between potassium intake and H-type hypertension, this study is hoped to spark academic interest in this area.

## 5 Conclusions

In our study, we found that 24-h urinary potassium excretion was negatively associated with the risk of both H-type hypertension and non-H-type hypertension, with a more stable negative response observed in H-type hypertension across logistic models and RCS curves. These findings suggest that H-type hypertension might be more sensitive to the diet - potassium excretion - Hcy metabolism - blood pressure regulation pattern, potentially due to its strong association with Hcy, which is influenced by potassium intake and vitamin B status. Therefore, we recommend that hypertension patients measure Hcy level at least once to identify whether they are H-type hypertension. In addition to the general drugs and lifestyle intervention, it is recommended that people with H-type hypertension strengthen the KAB on salt and hypertension, take initiative to reduce sodium salt intake, adopt reasonable dietary patterns and cultivate healthier dietary and lifestyle behaviors, so as to improve potassium intake, and ultimately aid in better Hcy and blood pressure management.

## Data Availability

The original contributions presented in the study are included in the article/Supplementary Material. Further inquiries can be directed to the corresponding author.
